# Transoral endoscopic thyroid lobectomy and ipsilateral central neck lymph node dissection vestibular approach: analysis of the learning curve and clinical outcomes evaluation

**DOI:** 10.3389/fendo.2024.1498797

**Published:** 2025-01-06

**Authors:** Yuhang Deng, Jiaojiao Zhao, Mei Tao, Haixin Zhao, Ruoxin Fan, Xiaoming Wang, Xiubo Lu

**Affiliations:** ^1^ Department of Thyroid Surgery, Zhengzhou University First Affiliated Hospital, Zhengzhou, Henan, China; ^2^ Oncology Department, Tianjin Medical University, Tianjin, China

**Keywords:** transoral endoscopic thyroidectomy vestibular approach, thyroid cancer, learning curve, thyroidectomy, minimally invasive surgery

## Abstract

**Purpose:**

The transoral endoscopic thyroidectomy vestibular approach (TOETVA) is distinguished by its ability to leave no visible scars on the body’s surface. Currently, there is still a lack of single-center large sample size analysis on the learning curve of TOETVA, especially for the treatment of thyroid cancer. This study aims to fill this void by presenting a comprehensive analysis of the learning curve and assessing the procedure’s feasibility in managing thyroid cancer.

**Methods:**

Between June 2020 and June 2023, a retrospective analysis was conducted on 195 patients who had undergone the transoral endoscopic thyroidectomy vestibular approach (TOETVA) at the First Affiliated Hospital of Zhengzhou University. We employed the cumulative sum method (CUSUM) to delineate the learning curve of TOETVA. Additionally, clinical parameters across different stages of the learning process were meticulously compared and analyzed.

**Results:**

All patients successfully completed endoscopic surgery without conversion to open surgery. Utilizing the CUSUM algorithm, two distinct learning phases were delineated: the exploration phase, comprising 58 cases, and the maturation phase, encompassing 137 cases. Analysis revealed that the maturation phase demonstrated significantly reduced operative times (189.7 ± 237.27 *vs*. 138.15 ± 26.62 minutes, p<0.001), decreased blood loss (15.49 ± 15.05 *vs*. 9.67 ± 4.12 ml, p=0.005), and a lower incidence of complications (7 *vs*. 4, p=0.028) when compared to the exploration phase. Additionally, in the maturation phase, we achieved successful surgical outcomes in a subset of obese patients and those with nodular goiter.

**Conclusion:**

TOETVA has been demonstrated to be safe and feasible, with the capability to effectively address complex cases once the learning curve has been surmounted.

## Introduction

In 2022, thyroid cancer became the fifth most common type of cancer in China, marked by the steepest average annual percentage increase and a pronounced predilection among women (1, 2). Open thyroidectomy remains the primary treatment for papillary thyroid carcinoma, with treatment guidelines mandating routine central lymph node dissection for this condition (3). However, this method results in a neck scar, significantly affecting appearance, life, work, and psychological stress, especially in patients prone to keloid formation (4–6). Endoscopic subtotal parathyroidectomy was initially reported in 1996 (7), and since then, multiple techniques, such as transthoracic, periareolar, and axillary approaches, have been developed to reduce scarring. Wilhelm pioneered the first endoscopic thyroidectomy through an oral approach (8), which, due to its associated complications, has been largely superseded by the three-port oral vestibular technique introduced by Wang Cunchuan (9). Studies by Anuwong, Guibin Zheng, and others have reported the safety and feasibility of this procedure (10–12). Recently, the transoral endoscopic thyroidectomy vestibular approach (TOETVA) has gained widespread attention owing to its unique perspective, shorter dissection path, minimal surgical trauma, and the notable benefit of being scarless on the body surface (13, 14).

Due to the lack of a natural orifice in the human body that directly leads to the thyroid, endoscopic thyroidectomy is a time-consuming and technically challenging procedure. Especially for TOETVA, compared with other endoscopic thyroidectomy approaches, it may have a longer learning curve due to its unique surgical perspective and the smaller operating space.

The learning curve refers to the process of gradually mastering and becoming proficient in skills through continuous learning. Analyzing the learning curve and summarizing the key points of learning and common complications associated with new technologies can facilitate the growth of clinical physicians’ experience. Currently, there is a lack of large-sample, single-center analyses concerning the learning curve for TOETVA, particularly in the treatment of malignant tumors. This study addresses this gap by investigating the learning curve of TOETVA and assessing the impact of mastering this technique on broadening its applicability. The study encompasses 195 cases, all performed by a single surgeon (XM-W), with postoperative pathology confirming all cases to be papillary thyroid carcinoma.

## Methods

### Study design

This retrospective analysis included clinical data from 227 TOETVA cases performed at the same center (the First Affiliated Hospital of Zhengzhou University) from June 2020 to June 2023. For the learning curve analysis, only cases that involved ‘unilateral thyroid lobectomy plus ipsilateral central neck lymph node dissection’ were considered. The surgeon had over a decade of experience in open thyroidectomy, six months of training at a pioneering medical center for TOETVA but no prior experience as the primary surgeon in endoscopic surgeries. Strict control over surgical indications was maintained in the early stages, prioritizing ideal cases. As the technique matured, more complex procedures were attempted. All patients underwent detailed preoperative communication and signed an informed consent form the surgery.

### Indications

(1) No oral infections confirmed preoperatively; (2) No prior neck surgeries; (3) No evidence of lateral neck lymph node metastasis or distant metastasis; (4) For malignant tumors, the maximum diameter does not exceed 2 cm; (5) Imaging assessments indicate that thyroid nodules are located away from vital structures such as the trachea and recurrent laryngeal nerve; and (6) Lesion localized to the lower pole of the thyroid gland.

### Preoperative preparation

All patients underwent standard preoperative assessments to ascertain their suitability for surgical intervention. The patient’s lips and mandible were assessed, and male patients had a submental area shaved in preparation for surgery. All patients received health education preoperatively and underwent oral hygiene with chlorhexidine solution one day prior to surgery. Prophylactic antibiotics were administered 30 min before surgery.

### Surgical procedures

**Figure 1:**
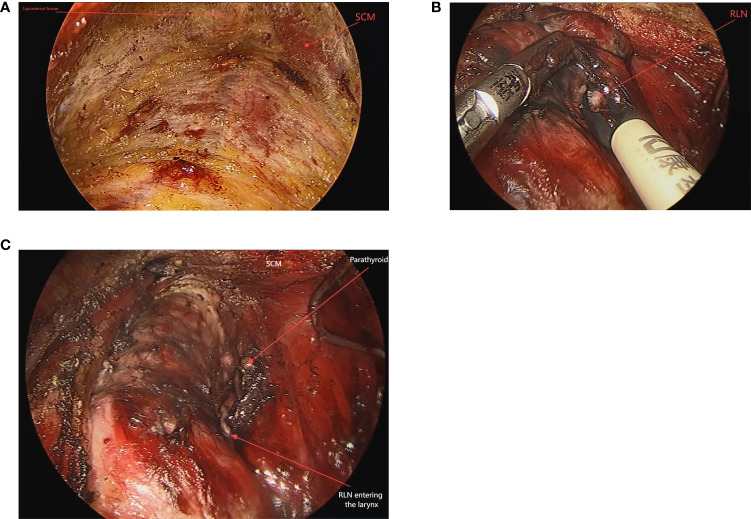
The operative space of TOETVA **(A)**. Assist in locating the recurrent laryngeal nerve from above downwards using a nerve detector, which also has the function of tissue dissection **(B)**. The surgical field after thyroidectomy **(C)**.

The patient was placed in a supine position with padding placed beneath the shoulders and neck to achieve slight hyperextension of the head, aligning it with the edge of the bed. General anesthesia and orotracheal intubation were administered. Sterile drapes and a protective skin film were used to cover and protect the area above the patient’s lips and the maxillofacial region. A horizontal incision of approximately 2 cm was made in the central part of the oral vestibule, followed by the insertion of a 10 mm trocar. CO_2_ gas was introduced, maintaining a pressure of 4 mmHg. Through this trocar, the endoscope was inserted. Incisions of about 5 mm were made on the buccal side of the lower cuspid tooth on both sides, and a 5 mm trocar was inserted along the lateral edge of the mandible to introduce the operative instruments. Under direct endoscopic vision, the space beneath the platysma muscle was dissected, extending laterally to the anterior border of the sternocleidomastoid muscles and inferiorly to the sternal notch, to establish the surgical space (**Figure 1A**). The isthmus and pretracheal space were separated using dissecting forceps. An ultrasonic scalpel was used to divide the tissue. The thyroid gland was separated between the true and false capsules, lifting the lobe, freeing the upper pole, and stepwise coagulating the superior artery. The superior pole of the thyroid was elevated ventrally, preserving the superior and inferior parathyroid glands *in situ*. The middle vein was identified, coagulated, and divided. The recurrent laryngeal nerve was carefully explored and identified, with measures taken to protect the nerve (**Figure 1B**). Continuous intraoperative nerve monitoring was performed. The suspensory ligament was divided and the thyroid gland was completely excised. The specimen was removed through a midline incision using a specimen retrieval bag. Along the carotid sheath on one side, ipsilateral level VI lymph nodes were carefully dissected from the surrounding tissue. The recurrent laryngeal nerve was meticulously protected, thymus was preserved, and lymph nodes were completely cleared (**Figure 1C**). The specimen was sent for pathological examination after ensuring that no parathyroid tissue was present. Pre- and postoperative signals from the recurrent laryngeal nerve monitor were compared to ensure nerve function. The surgical field was thoroughly checked for hemostasis and irrigation. The incision was closed with inverted sutures using non-absorbable suture material, and a negative-pressure suction device was placed through a submental route. The oral incision was then closed layer by layer with absorbable sutures.

### Postoperative management

Postoperatively, patients were instructed to continue rinsing their mouth with a compound chlorhexidine solution for one week. The drainage tube was removed if the drainage fluid appeared normal in color and the daily volume did not exceed 10 ml. Following tube removal, intravenous antibiotic administration was ceased, and the patient was observed for an additional day before discharge.

### Observation indexes

Operation time was defined as the period spanning from the start of surgery after anesthesia to the end of the surgery when anesthesia was stopped. The bleeding amount was estimated based on the number of blood-stained gauze pads used during surgery, with data extracted from the surgical records. Pathologic results, number of lesions, tumor size(for multifocal cases, only the largest diameter was recorded), thyroid gland volume, and the number of lymph nodes retrieved came from the routine postoperative pathology report. Demographic data of the patients, their postoperative hospital stay, and any postoperative complications were also recorded. The Cumulative summation(CUSUM) for the first patient was the difference between the first patient and the average surgery time of all selected patients. Subsequently, the CUSUM for the patients was included in the same manner as in the previous procedures, and then the CUSUM for all patients were accumulated and calculated to derive the learning curve.

### Statistical analysis

Descriptive statistics were used to summarize the data. For categorical variables presented frequency or percentage, the Fisher’s exact test or chi-square test was implemented as appropriate. As for continuous variables, Student’s t-test, Wilcoxon rank-sum test, ANOVA test, and the Kruskal-Wallis test were applied. The CUSUM procedure was used to fit the learning curve and the distinct learning stages were determined via the optimal inflection point. The significance level α was set to 0.05. All p-values were two-tailed unless otherwise depicted.

## Results

Between June 2020 and June 2023, a total of 227 TOETVA procedures were conducted by the same surgeon, with no conversions to open surgery. Of these, 195 patients who were preoperatively diagnosed with unilateral papillary thyroid carcinoma underwent ‘unilateral thyroid lobectomy plus ipsilateral central neck lymph node dissection’ and were included in the learning curve analysis. Additionally, 14 patients with nodular goiter underwent only unilateral thyroid lobectomy. In 18 patients, total bilateral thyroidectomy was performed, with or without central compartment lymph node dissection.

The demographic characteristics and surgical outcomes of all the patients included in the learning curve are detailed in **Table 1**. A total of 195 patients underwent endoscopic surgery without conversion to open surgery. Among these, 88 had left thyroid surgery and 107 had right thyroid surgery; 24 (12.3%) were male and 171 (87.7%) were female; the average age was 32.2 ± 6.67 years; and the average BMI was 22.96 ± 3.62 kg/m². The average operation time was 152.24 ± 39.02 minutes, and the average intraoperative blood loss was 11.41 ± 9.31 ml. Pathological results confirmed all cases as papillary thyroid carcinomas, with a lesion diameter of 0.56 ± 0.36 cm. Thyroid volume ranged from 4.8 to 65.62 ml; pathologically identified lymph nodes and positive lymph nodes were 4.42 ± 3.18/0.85 ± 1.37; A total of 11 surgical complications occurred in the patients included in the learning curve. One patient developed tracheal ulceration and was hospitalized for up to 30 days postoperatively; two patients developed cervical infection, with postoperative hospital stays of 11 and 18 days, respectively; two patients experienced temporary laryngeal recurrent nerve palsy, which recovered during the one-month postoperative follow-up; and six patients had postoperative numbness of the chin and lower lip, all of which resolved within six months. No patients developed hypoparathyroidism, which may be related to the fact that all procedures performed were unilateral thyroid lobectomy.

**Table 1:** Demographic characteristics and surgical outcomes of patients.

Variable	
**Age, years ^1^**	32.2 ± 6.67
Sex	
male	24 (12.3%)
female	171 (87.7%)
**Body mass index, kg/^2 1^**	22.96 ± 3.62
Unilateral thyroidectomy	
Left thyroidectomy	88 (45.1%)
Right thyroidectomy	107 (54.9%)
Pathology	
Papillary thyroid cancer	195 (100%)
**Operation time, min ^1^**	152.24 ± 39.02
**Bleeding amount,ml ^1^**	11.41 ± 9.31ml
**Thyroid volume,cm³**	4.80~65.62ml
Lesions number	
single	174 (89.2%)
multiple	21 (10.8%)
**Tumor size (largest), cm ^1^**	0.56 ± 0.36
Central lymph node dissection 1	
Retrieved	4.42 ± 3.18
Positive	0.85 ± 1.37
**Hospitalization, day ^1^**	3.19 ± 1.42
Complications	
Tracheal ulcer	1 (0.5%)
Surgical site infection	2 (1.0%)
RLN palsy	2 (1.0%)
Mental nerve injury	6 (3.1%)

**^1^** Data is presented as the mean ± standard deviation. RLN, recurrent laryngeal nerve.

**Figure 2:**
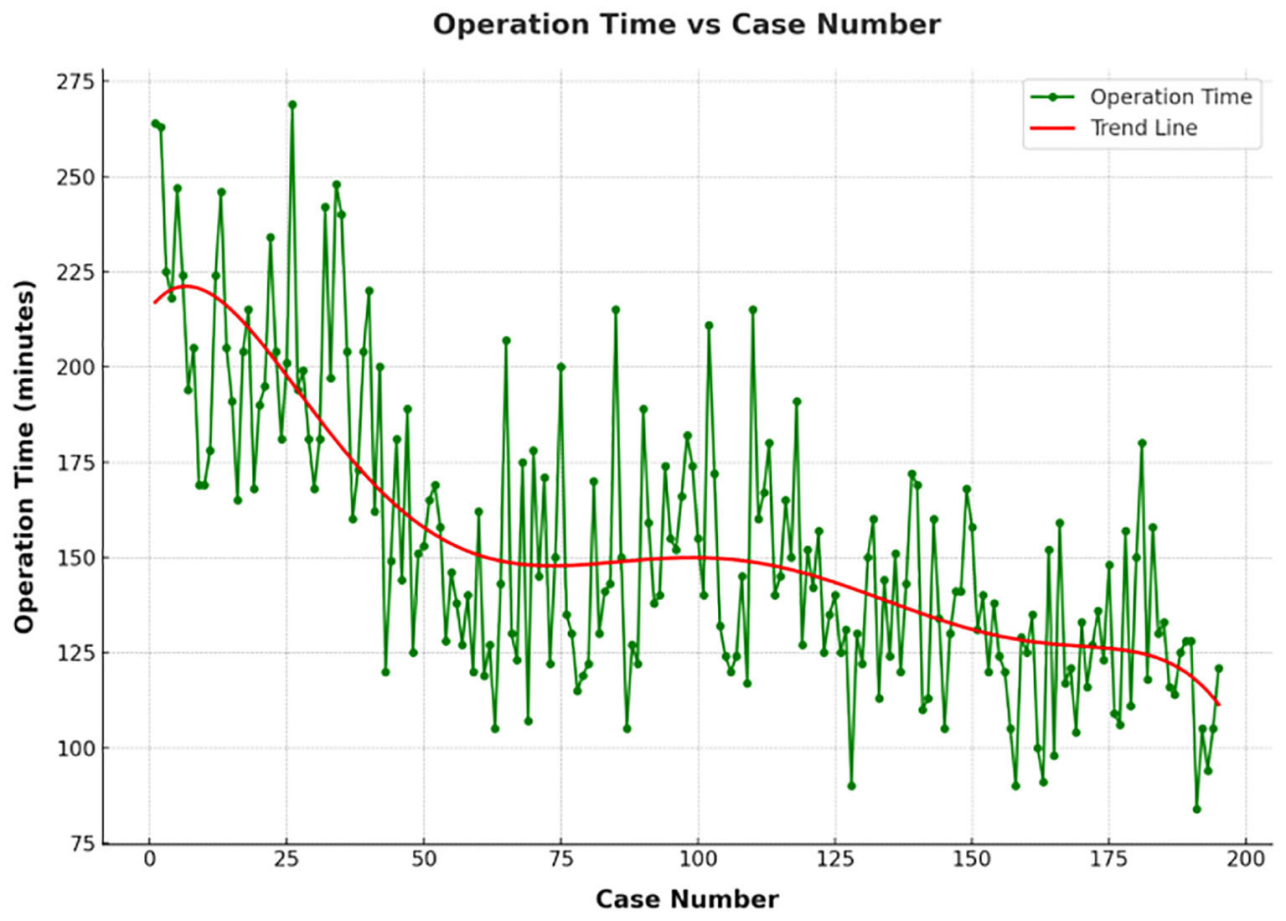
The operation times of 195 patients who received TOETVA, depicted in chronological order.

**Figure 3:**
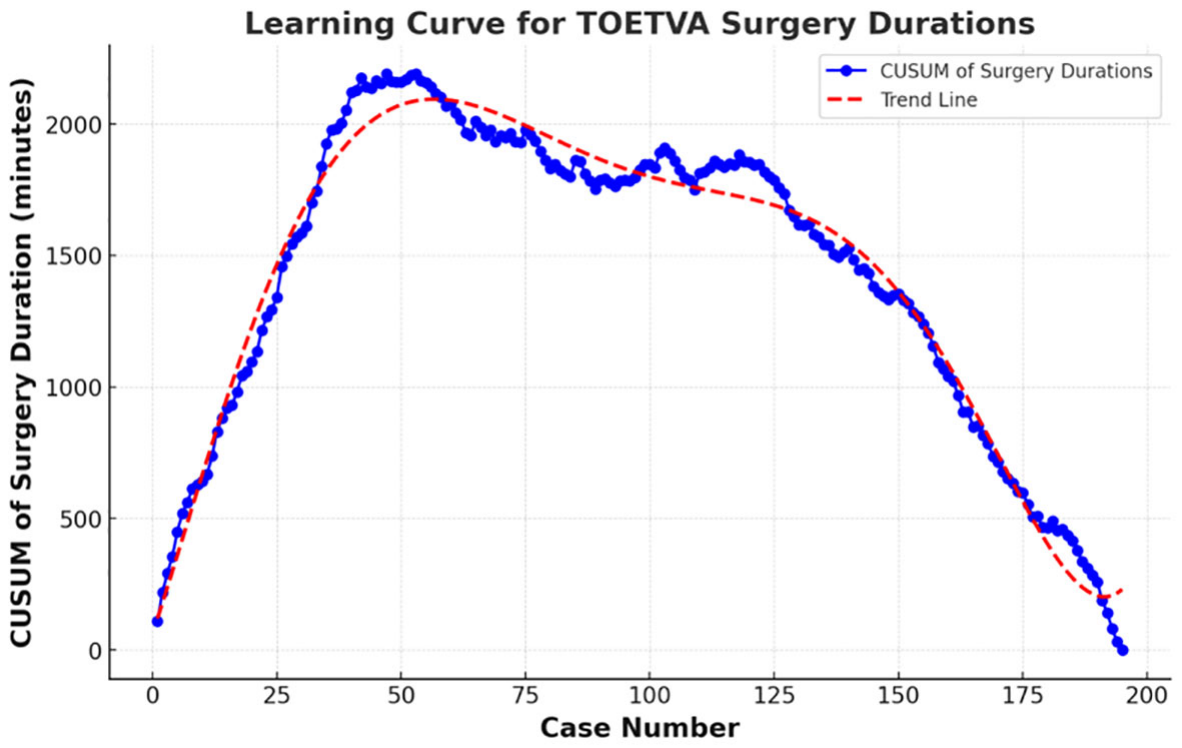
Learning curve for TOETVA using cumulative sum (CUSUM) method.

As depicted in the scatter plot of patient surgery times and bleeding amounts (**Figure 2**), there was a noticeable decrease in operation time with an increase in the number of surgical cases, accompanied by a trend towards reduced bleeding amounts. The best fit for the curve was a sixth-order polynomial with equation CUSUM equal to 4E-09*(Case number)^6^- 2E-06*(Case number)^5^+0.0004* (Case number)^4^- 0.0359*(Case number)^3^+ 0.6027*(Case number)^2^+58.908*(Case number)+ 56.778 (**Figure 3**), which had a high R2 value of 0.9786. The turning point on the learning curve was at the 58th case. Based on the learning curve, the initial 58 cases were designated as the technology exploration phase, while the following 137 cases constituted the technology maturity phase. The demographic characteristics and surgical outcomes for each phase are detailed in **Table 2**. Upon reaching the technology maturity phase, we completed surgeries for 13 obese patients and 14 patients with nodular goiter, without any complications. Further demographic characteristics and surgical outcomes are detailed in **Tables 3** and **4**.

**Table 2:** Demographic characteristics and surgical outcomes at different stages.

Variable	Exploring phase	Mature phase	P-value
**Age, years ^1^**	32.80 ± 7.32	32.00 ± 6.41	0.433^*^
Sex			0.947^**^
male	7	17	
female	51	120	
**Body mass index, kg/m^2^**	23.40 ± 3.49	22.79 ± 3.67	0.308^*^
Pathology			
Papillary thyroid cancer	58	137	
**Operation time, min ^1^**	189.72 ± 37.27	138.15 ± 26.62	<0.001^*^
**Bleeding amount, ml ^1^**	15.49 ± 15.05	9.67 ± 4.12	0.005^*^
**Thyroid volume, cm³ ^1^**	15.74 ± 9.24	18.25 ± 9.28	0.091^*^
**Lesions number ^1^**	1.11 ± 0.41	1.11 ± 0.31	0.972^*^
**Tumor size(largest), cm ^1^**	0.52 ± 0.32	0.58 ± 0.37	0.297^*^
Central lymph node dissection ^1^			
Retrieved	4.95 ± 3.56	4.2 ± 3.00	0.146^*^
Positive	1.00 ± 1.24	0.79 ± 1.43	0.331^*^
**Hospitalization, day ^1^**	3.44 ± 1.40	3.09 ± 1.52	0.148^*^
Complications	7	4	0.028
Tracheal ulcer	1	0	0.297^F^
Surgical site infection	2	0	0.087^F^
RLN palsy	1	1	0.507**^F^**
Mental nerve injury	3	3	0.516^F^

**^1^** Data is presented as the mean ± standard deviation. ^*^Student’s T-test. ^**^Chi-square test. ^F^Fisher’s Exact Test. RLN, recurrent laryngeal nerve.

**Table 3:** Demographics and surgical data of obese patients (n= 13).

Number	Age(years)	Sex(F/M)	BMI(kg/m²)	Operating time (min)	Bleeding amount (ml)	Central lymph node dissection		Hospitalization (day)
						Retrieved Positive		
1	34	F	29.30	181	10	14	0	4
2	26	M	37.871	215	30	2	1	4
3	29	F	28.84	174	5	9	1	3
4	43	F	28.23	172	5	3	2	5
5	40	M	29.05	215	10	8	2	4
6	20	F	29.74	150	5	4	1	2
7	29	M	29.41	130	10	4	1	4
8	23	M	28.96	135	10	1	0	2
9	22	F	28.24	100	10	5	4	2
10	43	F	28.26	152	5	8	0	3
11	31	M	28.72	136	5	2	0	2
12	42	F	28.04	148	10	2	0	2
13	28	F	36.05	130	15	1	0	2

F, female; M, male.

**Table 4:** Demographics and surgical data of patients with nodular goiter (n=14).

Number	Age (years)	Sex(F/M)	BMI (kg/m²)	Operation time (min)	Bleeding amount (ml)	Tumor size (The largest, cm)	Thyroid volume (cm³)	Hospitalization (day)
1	48	F	21.45	140	10	2.2	30	4
2	29	F	19.49	172	5	3.2	22.5	9
3	48	F	22.46	122	10	3.1	37.8	2
4	32	F	18.90	156	10	3.0	30	3
5	12	F	19.33	145	10	2.8	47.6	2
6	29	F	21.48	142	5	2.5	13.5	2
7	17	F	19.38	121	15	3.5	39.375	3
8	34	F	21.26	111	20	3.0	31.5	9
9	42	F	21.91	177	5	2.8	12.096	5
10	34	F	20.76	120	10	4.8	54.6	2
11	26	F	20.20	148	10	2.9	44.4	2
12	23	F	24.91	117	5	3.3	18	3
13	22	F	18.43	100	5	4.2	48.125	3
14	33	F	18.59	173	10	3.5	27	2

F, female; M, male.

## Discussion

TOETVA, being the sole method without a skin incision, has gained widespread adoption both domestically and internationally in recent years. However, due to its high technical difficulty, the surgeon needs a certain amount of study and practice to become proficient. Therefore, exploring the learning curve is of significant importance for training new surgeons and assessing the maturity of surgical skills, which helps guide surgeons to gradually improve their surgical techniques in practice. In this study, we explored the learning curve and the short-term postoperative outcomes of TOETVA.

Currently, there is a scarcity of large-sample single-center analyses regarding the learning curve for TOETVA. Reports on the learning curve for TOETVA vary among different centers and surgeons. For instance, Kuo et al. reported on 119 cases of TOETVA performed under neuromonitoring, concluding that surgeons without prior laparoscopic experience would require 35 cases to overcome the learning curve (15). Chai et al. reported 110 cases of TOETVA, determining that 57 cases were needed to overcome the learning curve (16). While these studies offer preliminary reference data, their small sample sizes may limit the accuracy of reflecting the actual situation. In this study, we included a total of 195 cases in the learning curve analysis, with postoperative pathology confirming all cases as papillary thyroid carcinoma. It is worth noting that in this center, a continuous series of 110 cases undergoing ‘unilateral thyroid lobectomy plus Ipsilateral central neck lymph node dissection’ from June 2020 to May 2022 were included in the learning curve analysis, leading to the conclusion that 38 cases are needed to overcome the learning curve and enter a phase of technical maturity. However, in this study, with a sample size of 195 cases, the inflection point of the learning curve was identified at the 58th case. Dividing the cases after entering the phase of technical maturity into two parts, we observed a continued gradual decrease in operation time(148.19 ± 26.94 *vs*. 127.97 ± 21.56, P<0.001). This indicates that expanding the sample size resulted in a decrease in the overall average operation time for that period. Using the cumulative sum method to fit the learning curve with all cases included delays the inflection point and increases the number of cases required to overcome the learning curve. This suggests that for research on the learning curve of TOETVA, a larger sample size and longer-term follow-up analysis are required to more accurately determine the critical moment when the surgeon masters the technique. In terms of surgical operations, there is a correlation between operation time and bleeding amount. With the accumulation of the surgeon’s experience, the bleeding amount decreases, which also leads to a decrease in the number of times the suction device is used to clean the surgical field, thereby shortening the operation time. Moreover, in this study, the first 80 cases applied the suspension technique to maintain space during the cavity construction process, while in subsequent surgical cases, the aspirator support technique was used. Specifically, the laparoscopic suction device was introduced laterally through the observation port to support the skin and muscle tissue above the surgical field, while also aspirating smoke from the surgical area. This technique also reduced surgery time and avoided the needle marks and indentations in the neck that occur early postoperatively with the use of suspension. Additionally, in male patients with a prominent Adam’s apple, surgical challenges may arise. Firstly, a prominent Adam’s apple can obstruct part of the surgical field due to the top-down perspective of TOETVA, increasing the difficulty of the surgery. Second, the prominent Adam’s apple itself can limit the mobility of surgical instruments.

In this study, 11 surgical complications occurred, and no cases developed hypoparathyroidism. However, the absence of hypoparathyroidism does not imply that the parathyroid glands were not injured during surgery. Even if the parathyroid gland on the operated side was injured, the normal function of the contralateral parathyroid gland could compensate, thus preventing the manifestation of hypoparathyroidism. Consequently, we are unable to ascertain the extent of parathyroid gland injury on the operated side.

Mental nerve injury is a complication specific to TOETVA that may lead to transient or permanent non-painful numbness of the lower lip and chin (17). We observed a total of 6 cases of transient numbness in the chin and lower lip, with 3 cases occurring during the technical exploration phase and 3 cases during the technical maturity phase, all of which resolved within 6 months postoperatively. To minimize damage to the main trunk of the mental nerve, this center places the operative port incision at the labial buccal mucosa just lateral to the second lower premolars bilaterally. There are also reports of actively dissecting the mental nerve to avoid nerve damage (18). However, anatomical variations in the mental nerve exist (19), which can influence the surgical approach.

The incision for TOETVA is located within the oral cavity, categorizing it as a Class II incision, which increases the risk of infection. Therefore, antibiotics should be routinely used for infection prophylaxis in TOETVA (20). In our center, prophylactic antibiotics are administered 30 minutes prior to surgery, and iodophor gauze is used intraoperatively to disinfect the oral cavity. Postoperatively, antibiotics are administered intravenously until the removal of the drainage tube. We observed two cases of infectious complications during the technical exploration phase, with postoperative hospital stays of 11 and 18 days, respectively.

Additionally, some complications may not occur in early cases, possibly because when a technique is first introduced, surgeons tend to select the most ideal cases.

Surgery in obese patients is considered challenging, hence during the technical exploration phase, we did not select obese patients (BMI > 28) for surgery. In the phase of technical maturity, we successfully performed surgery on a total of 13 obese patients, with an average BMI of 30.06 ± 3.13 kg/m² and the highest BMI being 37.87 kg/m². Comparative analysis of operation times revealed that the average operation time for obese patients (155.62 ± 34.74 minutes) was significantly longer than that for non-obese patients (136.27 ± 24.81 minutes), with the difference being statistically significant (P < 0.05). According to the surgeon’s description, obese patients tend to have more cervical fat accumulation and a thicker subcutaneous layer, which increases flap tension and makes it more difficult to identify the anatomical planes, thereby increasing the difficulty of establishing the operative space. During thyroidectomy, fat accumulation can alter the anatomical position of the thyroid gland and its surrounding tissues, further complicating the surgical procedure. None of the patients experienced complications. Although one patient had a higher volume of drainage postoperatively, which may have been due to fat liquefaction, this did not result in an increased length of hospital stay, which is consistent with the findings published by Anuwong (10). Furthermore, there are also reports of successful implementation of the TOETVA in patients with a BMI > 40 (21). However, given that obesity can prolong surgery duration, it is recommended that novices accumulate relevant experience with the TOETVA before operating on obese patients to ensure patient safety and the effectiveness of the surgery.

Upon reaching the phase of technical maturity, we successfully performed surgery on 14 patients with nodular goiter, with the largest lesion measuring 4.8 cm in diameter. This indicates that as our experience grows, the surgical indications are progressively expanded. However, for lesions with larger diameters, their volume may exceed the diameter of our observation ports, making the removal of the specimens relatively difficult. When analyzing the operation times, we found no statistically significant difference between the average operation time for patients with nodular goiter (139.43 ± 24.87 minutes) and those with malignant tumors (138.08 ± 26.35 minutes, P=0.855). We did not perform prophylactic central lymph node dissection for patients with nodular goiter, which may account for the minimal difference in operation time compared to patients with malignant tumors.

The number of lymph nodes dissected is often considered an important indicator for evaluating the efficacy of thyroidectomy under endoscopy. TOETVA has been shown to be as effective as the gold standard open surgery (22), and may even be more effective (23). However, the feasibility and safety of dissection for lateral cervical lymph nodes are still under exploration. Dissection of lateral cervical lymph nodes is more challenging than that of the central compartment lymph nodes due to the involvement of more complex anatomical structures and requirement for a broader surgical field. In summary, the top-down perspective of TOETVA provides a significant advantage for the dissection of zone IV, and experienced surgeons are also capable of performing zone III dissections. However, dissection of zone II remains challenging (24). Individual centers have initiated combined sternocervical and lateral cervical dissections using the transoral approach as a supplement to the transthoracic approach for lateral cervical dissection (25). A significant number of cases have confirmed its safety and feasibility, with a learning curve of 11 cases (23). Our center has also conducted combined transthoracic and transoral endoscopic surgery for thyroid cancer with lateral cervical lymph node dissection in a female patient with a BMI of 23.43. Preoperatively, metastasis to the right lateral cervical lymph nodes was confirmed, and the patient strongly preferred endoscopic surgery. After thorough evaluation of imaging and pathological examinations and a detailed explanation of the surgical risks, a ‘total thyroidectomy + right central neck dissection + right lateral neck dissection’ was performed. The surgery was completed successfully, with a total duration of 308 minutes, and the patient was hospitalized for 6 days postoperatively. No complications such as hypoparathyroidism or hoarseness occurred, and adjuvant therapy with iodine-131 was administered one month later. The short-term outcomes were satisfactory.

This study enrolled 195 patients who received TOETVE treatment. Based on the CUCUM procedure, we identified 58 cases as the optimal point for achieving proficient skill improvement. The TOETVA is a safe and feasible technique that can be performed within an acceptable operation time, with the average operation time continuing to decrease even during the technical maturity phase. With sufficient experience, surgeons can gradually expand the indications for surgery and successfully complete complex cases. Despite the study’s limitations, including its retrospective design and short-term follow-up, TOETVA emerges as a promising scarless procedure for thyroidectomy. Future research should focus on long-term outcomes to solidify its therapeutic role in endoscopic thyroid surgery.

Our study provides valuable information on the learning curve of the TOETVA, yet it is not without its limitations. For instance, being a single-center retrospective analysis, it may be subject to selection and recall bias. Additionally, we did not stratify the operation time into specific phases, such as cavity construction time, gland resection time, and suturing time. Lastly, although our study included over 200 cases of malignant tumors, the short-term follow-up did not permit an assessment of long-term oncological outcomes. Long-term follow-up studies are necessary to evaluate the safety and benefits of TOETVA.

## Data Availability

The raw data supporting the conclusions of this article will be made available by the authors, without undue reservation.
